# Pericyte-derived exosomal miR-210 improves mitochondrial function and inhibits lipid peroxidation in vascular endothelial cells after traumatic spinal cord injury by activating JAK1/STAT3 signaling pathway

**DOI:** 10.1186/s12951-023-02110-y

**Published:** 2023-11-27

**Authors:** Peng Gao, Jiang Yi, Wenjun Chen, Jun Gu, Sheng Miao, Xiaowei Wang, Yifan Huang, Tao Jiang, Qingqing Li, Wei Zhou, Shujie Zhao, Mengyuan Wu, Guoyong Yin, Jian Chen

**Affiliations:** 1 Department of Orthopedic, the First Affiliated Hospital of Nanjing Medical University, No. 300 Guangzhou Road, Nanjing, 210029 People’s Republic of China; 2 Department of Orthopedic, Changzheng Hospital, No. 415 Fengyang Road, Shanghai, 200003 People’s Republic of China; 3 Department of Orthopedic, Wuxi Xishan People’s Hospital, No. 1128 Dacheng Road, Wuxi, 214105 People’s Republic of China; 4 Department of Orthopedic, Suqian First People’s Hospital, No. 120 Suzhi Road, Suqian, 223812 People’s Republic of China; 5 Department of Orthopedic, Maanshan People’s Hospital, No. 45 Hubei Road, Maanshan, 243000 Anhui People’s Republic of China

**Keywords:** Exosomes, miRNA, Pericytes, Spinal cord injury, Mitochondrion, Lipid peroxidation

## Abstract

**Background:**

Spinal cord injury (SCI) remains a significant health concern, with limited available treatment options. This condition poses significant medical, economic, and social challenges. SCI is typically categorized into primary and secondary injuries. Inflammation, oxidative stress, scar formation, and the immune microenvironment impede axon regeneration and subsequent functional restoration. Numerous studies have shown that the destruction of the blood–brain barrier (BBB) and microvessels is a crucial factor in severe secondary injury. Additionally, reactive oxygen species (ROS)-induced lipid peroxidation significantly contributes to endothelial cell death. Pericytes are essential constituents of the BBB that share the basement membrane with endothelial cells and astrocytes. They play a significant role in the establishment and maintenance of BBB.

**Results:**

Immunofluorescence staining at different time points revealed a consistent correlation between pericyte coverage and angiogenesis, suggesting that pericytes promote vascular repair via paracrine signaling. Pericytes undergo alterations in cellular morphology and the transcriptome when exposed to hypoxic conditions, potentially promoting angiogenesis. We simulated an early ischemia-hypoxic environment following SCI using glucose and oxygen deprivation and BBB models. Co-culturing pericytes with endothelial cells improved barrier function compared to the control group. However, this enhancement was reduced by the exosome inhibitor, GW4869. In vivo injection of exosomes improved BBB integrity and promoted motor function recovery in mice following SCI. Subsequently, we found that pericyte-derived exosomes exhibited significant miR-210-5p expression based on sequencing analysis. Therefore, we performed a series of gain- and loss-of-function experiments in vitro.

**Conclusion:**

Our findings suggest that miR-210-5p regulates endothelial barrier function by inhibiting JAK1/STAT3 signaling. This process is achieved by regulating lipid peroxidation levels and improving mitochondrial function, suggesting a potential mechanism for restoration of the blood-spinal cord barrier (BSCB) after SCI.

## Background

Traumatic spinal cord injury (SCI) has severe consequences on patients' physical and mental health and professional development [1]. Patients with SCI frequently experience long-term sensory, motor, and neurological impairments as a result of both initial damage and subsequent complex secondary injury cascade [2, 3]. Primary injury causes immediate mechanical damage to the medulla spinalis, including damaged neurons and disruption of the blood-spinal cord barrier. The secondary injury cascade involves inflammation, cytotoxicity, and cell death [4, 5]. Following SCI, there is a rapid disruption of the microvascular system in the spinal cord, resulting in the infiltration of inflammatory cells and cytokines [6–8]. Moreover, bleeding progressively worsens spinal cord swelling, thereby exacerbating mechanical compression of the cord. Secondary damage often surpasses the extent of the primary injury.

The blood spinal cord barrier (BSCB) is an important interface between the medulla spinalis and the peripheral vascular system. It consists of astrocytes, endothelial cells, and pericytes, which coordinate the exchange of substances between the blood and the spinal cord [9]. Endothelial tight junctions serve as physical barrier [10–13], preventing the entry of macromolecules, cells, and harmful substances into the medulla spinalis [14, 15]. Pericytes reside in the interstitial spaces between endothelial cells, astrocytes, and neurons. In addition to receiving signals from adjacent cells [16–18], these cells maintain the integrity of the blood–brain barrier (BBB) [19–21]. Multiple studies have demonstrated that disruption of the BSCB after SCI can lead to secondary damage, including hemorrhage, peroxidation, and excessive inflammation [22]. Enhancing repair and maintaining the integrity of the BSCB can promote functional improvement and tissue repair after SCI.

Extensive research has been conducted over the past few decades on the effects of astrocytes and endothelial cells on BSCB. Related therapeutics primarily aim to protect specific cells or structures to minimize the subsequent damage [23, 24]. Pericytes are crucial for the development, preservation, and regulation of BBB. Impaired crosstalk between endothelial cells and pericytes can lead to dysfunction of the BBB following SCI [25, 26]. Pericytes enhance the paracellular barrier of brain endothelial cells [27, 28]. Moreover, pericytes mediate angiogenesis, microvascular stability, and vascular architecture during CNS development and repair [18, 29]. Following SCI, pericytes segregate from blood vessels and migrate towards the core of the developing fibrous scar, promoting healing of the lesion [30, 31]. Additionally, other studies have demonstrated the importance of pericytes in vascular remodeling following SCI [32]. The function of pericytes is complicated, and our understanding of the crosstalk between pericytes and endothelial cells is limited.

Exosomes are nano-sized liposomes that are released by cells and contain non-coding RNAs, lipids, proteins, and cytokines [33]. They mediate intercellular communication [34], regulate cellular functions, and influence pathological and physiological processes in various diseases [34–36]. Exosomes have emerged as a promising therapeutic strategy in recent years because of their advantageous characteristics, such as reduced adverse effects and ability to cross the BBB [37]. Following SCI, the spinal cord experiences a state of hypoxia, characterized by reduced oxygen concentration compared to its natural physiological conditions. In this environment, there is a significant increase in the quantity of exosomes released by cells, accompanied by alterations in their composition and content.

Glucocorticoids are among the few effective therapies for neuroprotection after acute SCI. High-dose glucocorticoid therapy can improve neurological recovery in patients with SCI by inhibiting lipid peroxidation during secondary damage [38]. Research suggests that microvascular disruption, which is crucial for progressive degeneration and the resulting functional deficits [39, 40], is partially attributed to microvascular ROS and lipid peroxidation [39]. Therefore, inhibiting lipid peroxidation in vascular endothelial cells following SCI and improving mitochondrial dysfunction are crucial.

This study aimed to investigate the paracrine influence of pericytes on endothelial cells following SCI considering the unique association between these two cell types. Our study demonstrated that exosomal miR-210 inhibits lipid peroxidation and protects mitochondrial function in the vascular endothelial cells of the spinal cord following injury. This improves BBB integrity via the JAK1/STAT3 pathway. This finding may further contribute to our understanding of the intricate interplay between pericytes and endothelial cells following SCI, and provide a potential therapeutic target for SCI.

## Results

### Increased permeability, decreased coverage, and disrupted tight junctions (TJs) in the BSCB after SCI

**Fig. 1:**
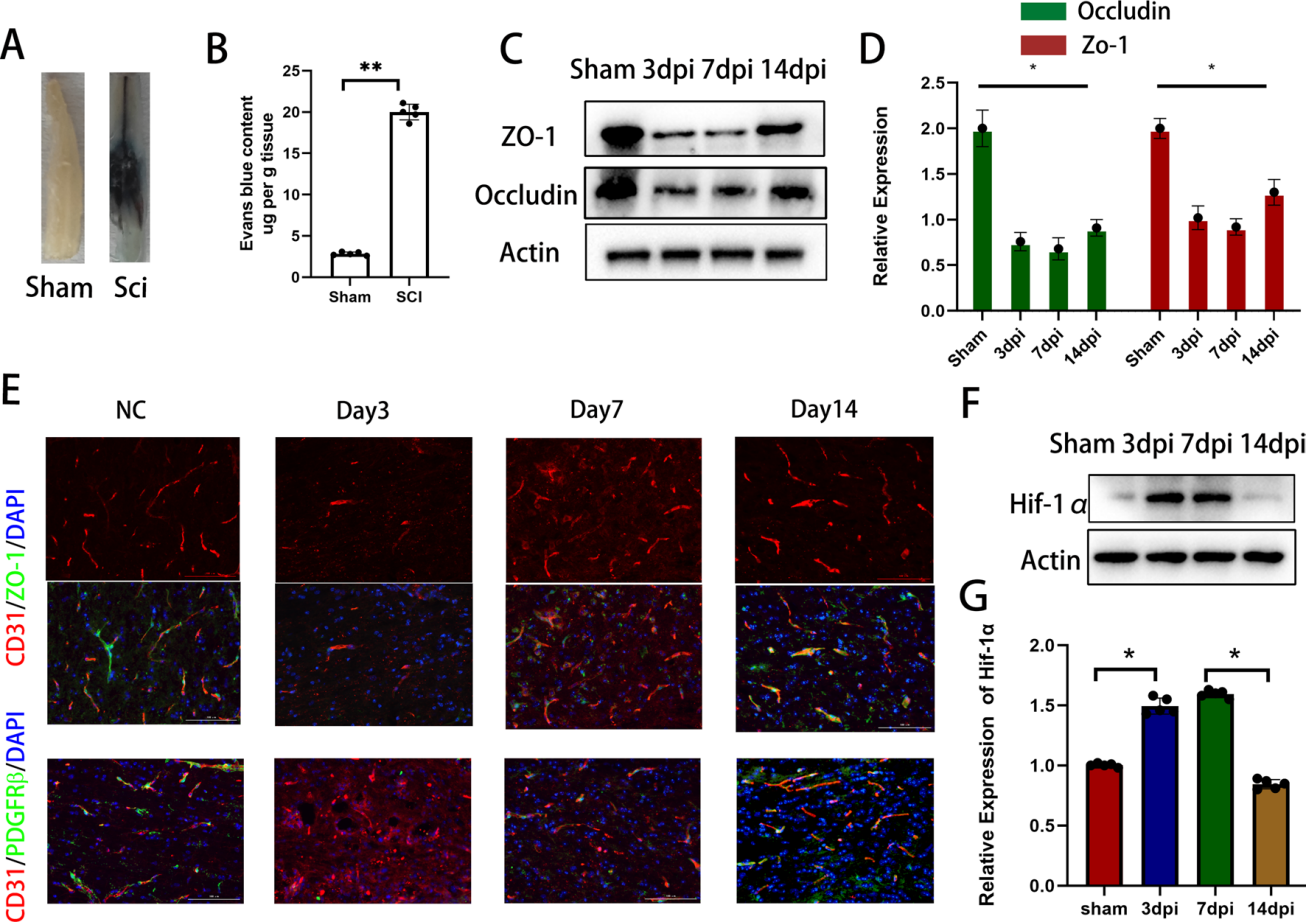
Increased permeability, decreased coverage, and disrupted TJs of BSCB after SCI. **A** Representative images of the spinal cord following EB injection 7 days after spinal cord injury. **B** Quantitative measurement of EB content in spinal cord injury areas. **C**, **D** Detection of TJs protein expression and quantification at different time points after SCI by immunoblotting. **E** Representative immunostaining of CD31, Pdgfrβ and TJs markers in the spinal cord injury area at different time points after SCI. **F**, **G** Expression levels and quantification of hypoxia-related proteins in spinal cord injury regions at different time points after SCI detected by immunoblotting

Following SCI, the integrity of the BBB was compromised. Figure 1a, b demonstrate prominent infiltration of EB dye in the spinal cord of the injured group, accompanied by a significantly higher EB content compared to the sham-operated group (Fig. 1a, b). Furthermore, following SCI, the protein expression levels of TJs in the injured spinal cord area were significantly reduced and only partially recovered after 14 days (Fig. 1c, d). Immunostaining of the injured site at different time intervals following SCI revealed that the blood vessels were primarily damaged during the early stages. The remaining blood vessels exhibited reduced colocalization with TJs, indicating an effect on the TJs of spinal cord microvessels during that time. At this time, pericyte coverage was extremely low (Fig. 1e). Over time, the expression of tight junctions in the blood vessels gradually increased, and the coverage rate of pericytes also increased synchronously. Furthermore, the hypoxic conditions following SCI did not show improvement until 14 days post-injury (Fig. 1f). Pericytes are intricately involved in the function of the blood-spinal cord barrier, suggesting their potential importance in promoting BSCB repair in a hypoxic environment following SCI.

### Uptake of pericyte-derived exosomes by endothelial cells

**Fig. 2:**
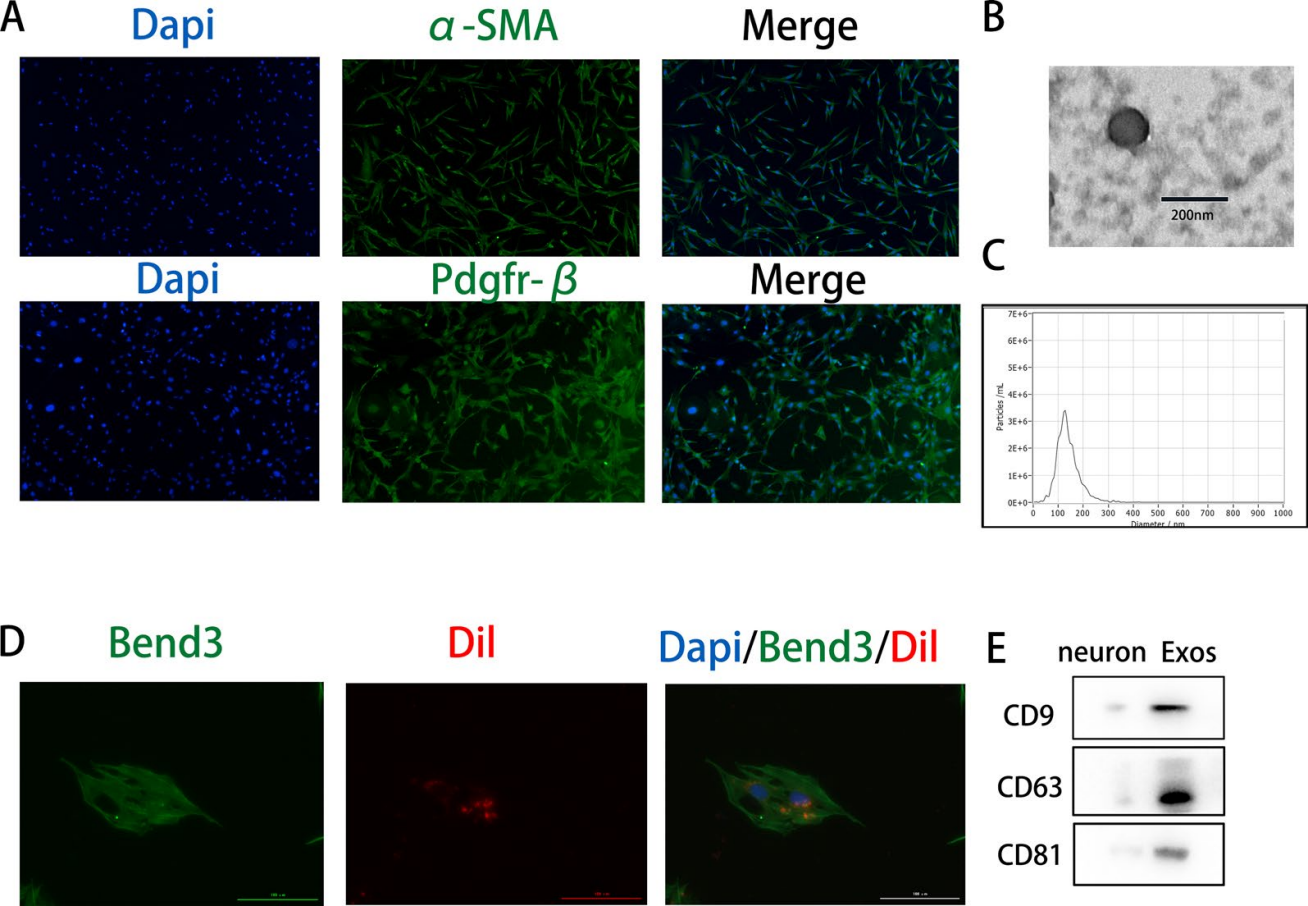
Endothelial cells uptake pericyte-derived exosomes. **A** Pericytes are identified by α-SMA and Pdgfrβ immunostaining. **B** Morphology of exosomes under fluoroscopic electron microscopy. **C** Determination of exosome particle size by NTA analysis. **D** Red fluorescent dye Dil-labeled exosomes are taken up by Bend3 endothelial cells. **E** Western blot analysis of exosome surface marker proteins

Primary pericytes were identified using immunofluorescence staining for α-SMA and PDGFRβ, both of which are pericyte markers (Fig. 2a). Exosomes were isolated from pericyte culture supernatants through a series of centrifugation steps and identified using NTA, TEM, and Western blot analysis. TEM revealed the morphology of exosomes (Fig. 2b). NTA showed that the nanoparticles typically had diameters between 50 and 150 nm (Fig. 2c). Western blot analysis revealed the presence of typical exosomal surface markers, including CD9, CD63, and CD81 (Fig. 2e). The uptake of Dil-labeled exosomes by endothelial cells was visualized using fluorescence microscopy (Fig. 2d).

### Pericyte-derived exosomes enhance endothelial cell barrier integrity in vitro

**Fig. 3:**
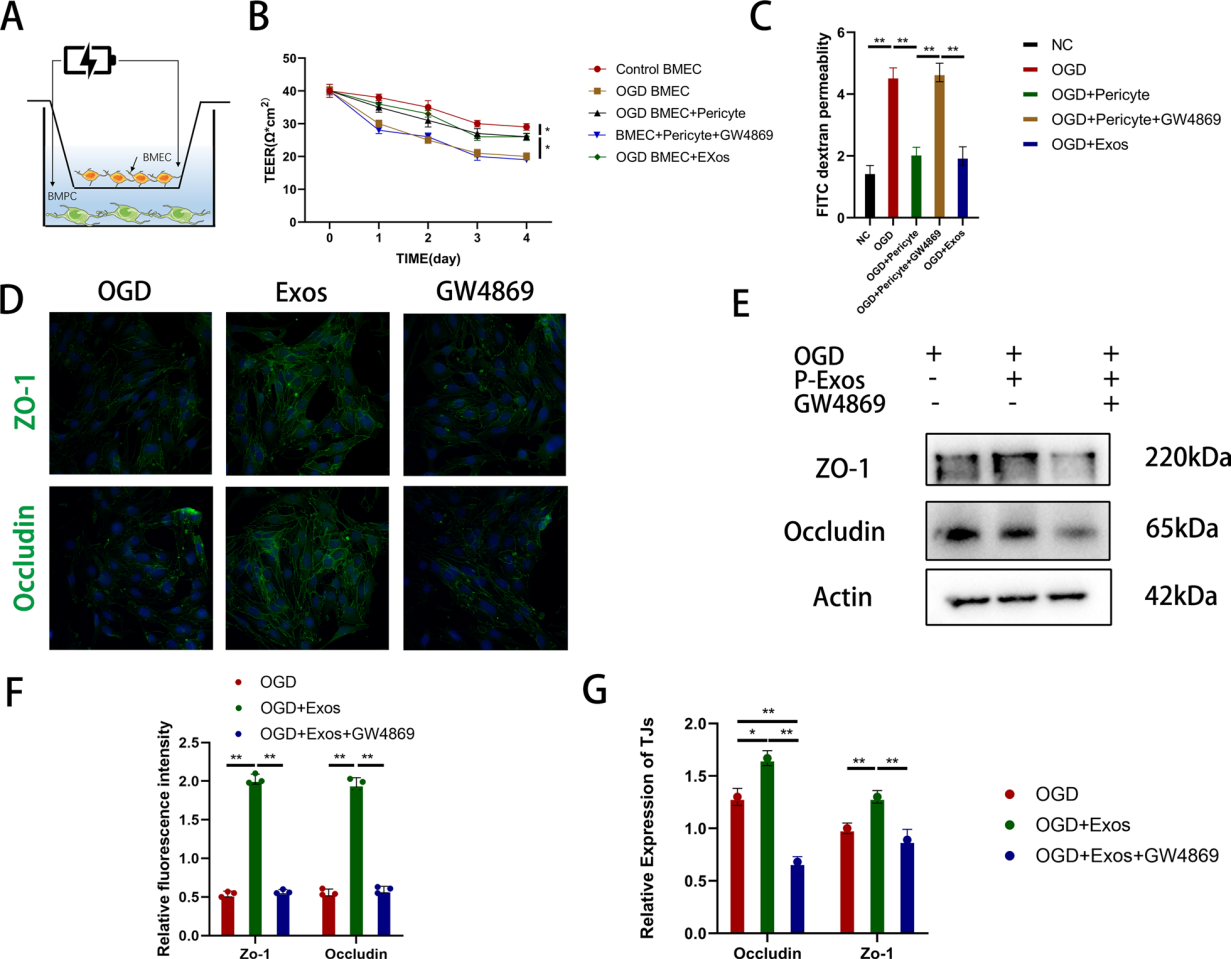
Pericyte-derived exosomes enhance endothelial cell barrier integrity in vitro. **A** Schematic diagram of in vitro blood–brain barrier model and measurement of TEER value. **B**, **C** TEER value and FITC-dextran permeation assay to evaluate the barrier function of Bend3 cells under different experimental conditions. **D**, **F** Immunofluorescence detection and quantification of ZO-1 and occludin protein expression levels in Bend3 cells. **E**, **G** Detection and quantification of TJs protein expression levels in Bend3 cells by western blotting

Next, we evaluated the effects of pericyte-derived exosomes on Bend3 cells cultured in vitro. OGD significantly reduced Teer values in normally cultured endothelial cells. However, co-culturing pericytes with Bend3 cells significantly mitigated the effect of OGD on their Teer values. We investigated the potential impact of pericytes on the underlying cells by administering pericyte-derived exosomes to OGD-exposed Bend3 cells. The results demonstrated comparable effects between the two conditions. Additionally, we used the exosome secretion inhibitor GW4869 and observed a partial reversal of this effect (Fig. 3a, b). Similar results were observed in the FITC-dextran permeability experiments (Fig. 3c). Immunostaining revealed that exosome treatment significantly increased the expression of Z0-1 and Occludin in Bend3 cells after injury, which was reversed by GW4869 (Fig. 3d, f). Similar findings were observed with the expression levels of TJs (Fig. 3e, g). We speculated that exosomes facilitate intercellular communication between pericytes and endothelial cells, thereby potentially contributing to the restoration of damaged endothelial cells.

### Pericyte-derived exosomes promote the recovery of motor function and protect the BSCB in mice after SCI

**Fig. 4:**
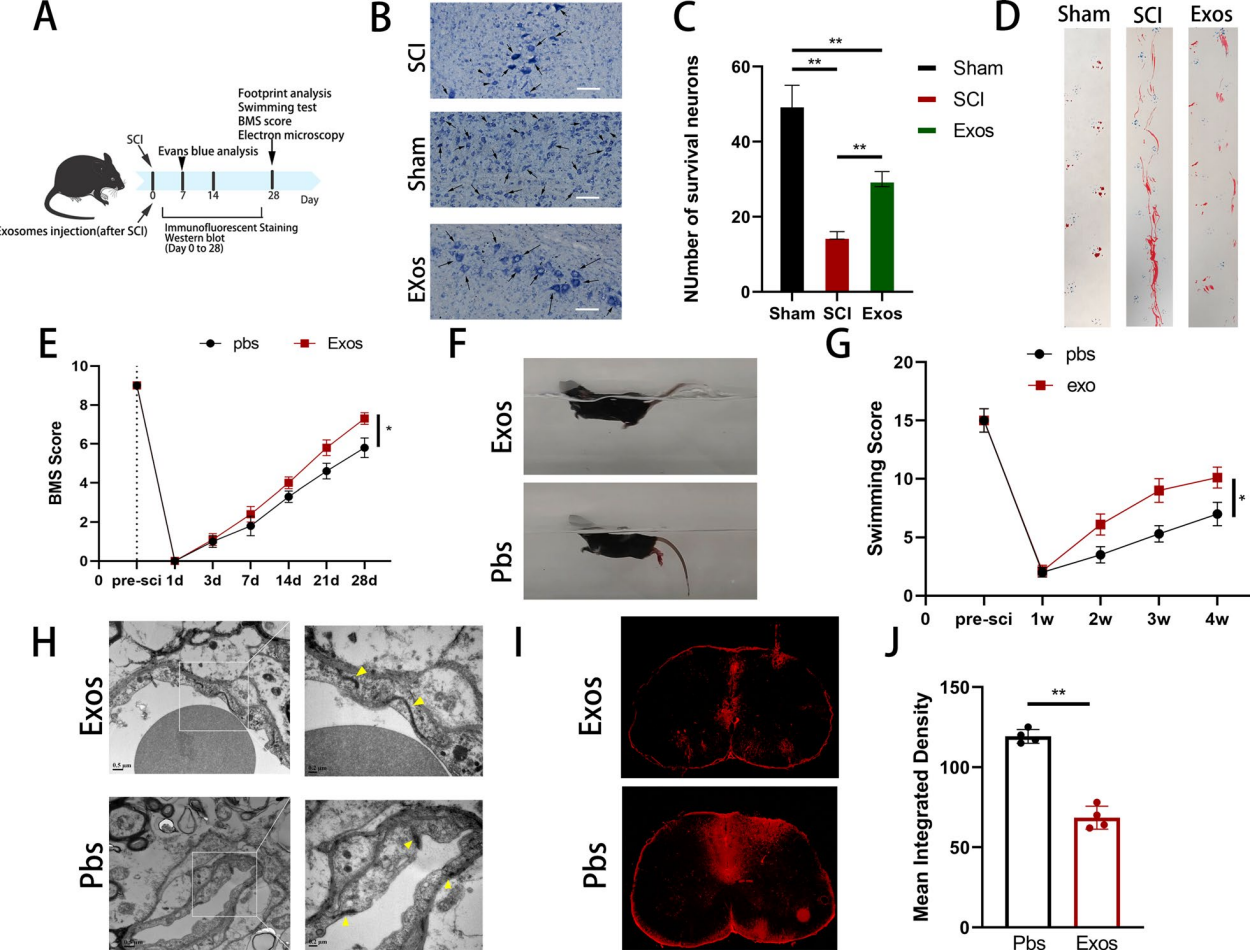
Pericyte-derived exosomes promote the recovery of motor function and protect BSCB in mice after spinal cord injury. **A** Schematic diagram of the experiment after spinal cord injury in mice. **B**, **C** Nissl staining showed and quantified the number of mouse motor neurons in each group. **D** Representative footprints of walking 28 days after spinal cord injury and quantifying the results of footprint analysis in individual mice. Blue: Forefoot; Red: Backfoot. **E** Functional classification of mice in each group using BMS scores from pre-injury to 28 days post-injury. **F**, **G** Functional grading at 28 days post-injury using the Louisville Swim scale and representative images. **H** The morphology of vascular tight junctions on the 28th day after spinal cord injury in different groups of mice was observed by transmission electron microscope. **I**, **J** Representative fluorescent images and quantification of spinal cord cross-sections in the injured area after EB injection at 7 days post-injury

We isolated pericyte-derived exosomes to investigate the effect of pericytes on vascular endothelial cells in vivo after SCI. These exosomes were then injected into mice following SCI. Subsequently, we performed relevant experiments at specific time intervals (Fig. 4a). Nissl staining served as a morphological indicator of neuronal functional activity. Seven days post-SCI, Nissl staining was performed to assess the neuronal count near the injury site. Our observations revealed that the exosome injection group exhibited a significantly higher number of neurons than the control group, but fewer than that in the sham-operated group (Fig. 4b, c). Furthermore, footprint experiments demonstrated that mice injected with pericyte-derived exosomes exhibited improved gait recovery compared with controls following SCI (Fig. 4d). Similar results were observed for the BMS score and swimming test (Fig. 4e–g). The density of vascular tight junctions was significantly higher in the exosome injection group than in the PBS group, as observed using TEM. In addition, the intercellular space appeared to be more closely connected (Fig. 4h). Following EB injection, spinal cord cross-sectional fluorescence images of the exosome group showed reduced EB dye penetration compared with the PBS group. These findings suggest that pericyte-derived exosomes may promote restoration of the BSCB and accelerate functional recovery following SCI (Fig. 4i, j).

### MiR-210-5p is upregulated after SCI and can be transferred into endothelial cells via exosomes

**Fig. 5:**
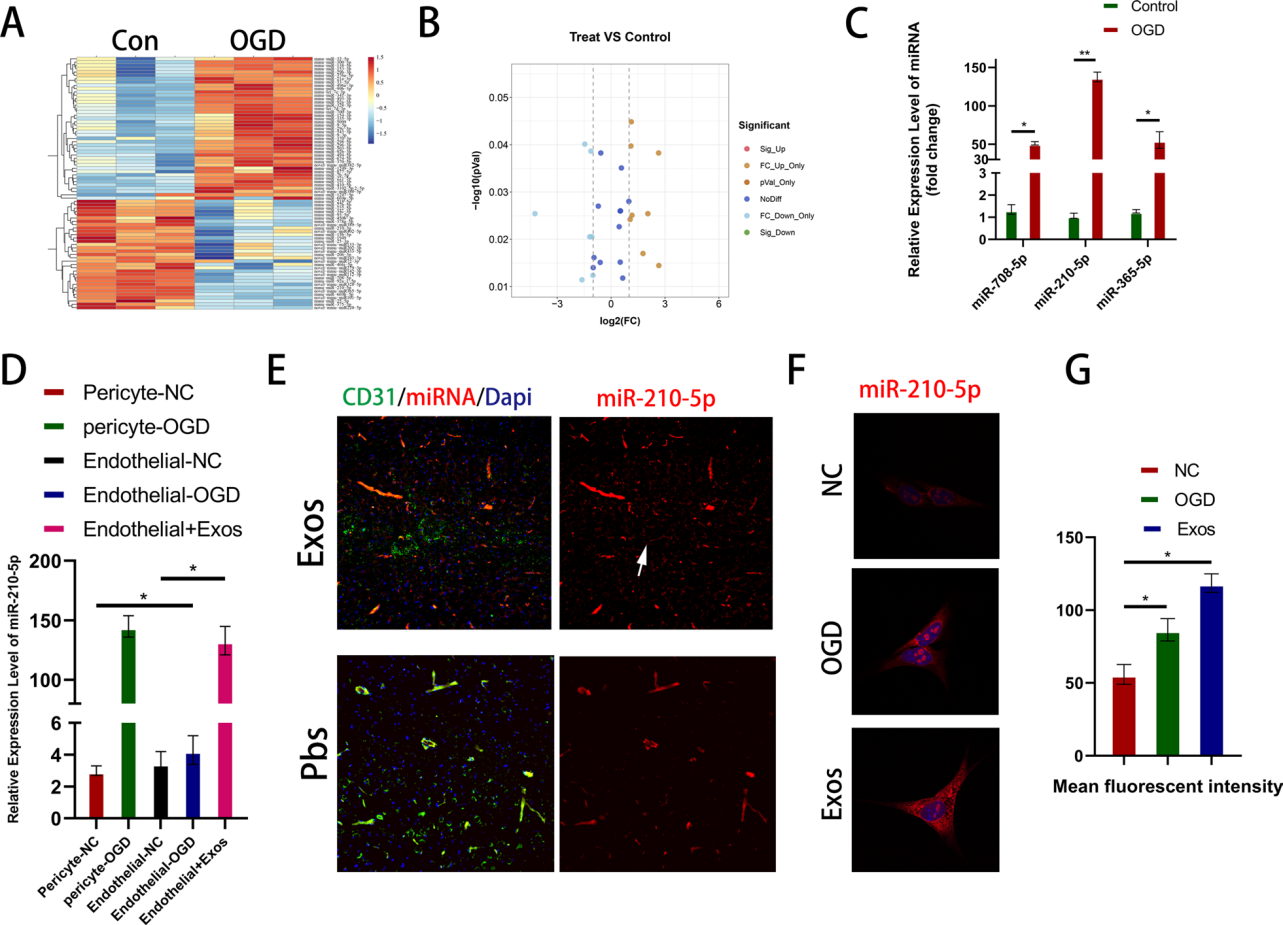
MiR-210-5p is upregulated after spinal cord injury and can be transferred into endothelial cells via exosomes. **A** Heatmap of miRNAs from differently treated pericytes. **B** 8 up-regulated miRNAs and 7 down-regulated miRNAs (FC threshold 2, *P* value 0.05). **C** The expression levels of miR-708-5p, miR-210-5p and miR-365-5p in endothelial cells were compared by qRT-PCR method. **D** Expression levels of miR-210-5p in pericytes and endothelial cells under different treatment conditions by qRT-PCR. **E**–**G** Detection and quantification of miRNA expression levels under in vitro and in vivo conditions by in situ hybridization

Previous in vivo and in vitro experiments have indicated that pericyte-derived exosomes can improve the barrier function and accelerate functional recovery. Multiple studies have demonstrated the crucial role of miRNAs in exosomes, mediating cell-to-cell communication and regulating target cells to execute distinct biological processes. Therefore, we simulated the ischemia-hypoxic environment following SCI using OGD. We extracted RNA from pericytes in the different treatment groups, sequenced miRNAs, and compared the differences between the two groups (Fig. 5a). The sequencing results revealed that 33 miRNAs were upregulated and 43 were downregulated in the OGD-exposed group compared to the control group (Fig. 5b). Next, we screened the miRNAs based on their expression differences using a volcano plot. Based on miRNA profiling data, we selected three miRNAs, miR-710-5p, miR-210-5p, and miR-365-5p, based on their high basal expression levels and significant upregulation. We used qRT-PCR in vitro to validate their expression. Based on the sequencing and qRT-PCR results, we focused on the most differentially expressed miRNA, miR-210-5p (Fig. 5c). qRT-PCR and in situ hybridization techniques were used to investigate the potential transfer of miR-210-5p by exosomes and its effects on endothelial cells. After OGD, pericytes exhibited a significant increase in miR-210-5p expression compared to that in the control group, whereas endothelial cells did not show similar results (Fig. 5d). Next, we co-stained the frozen sections with an miR-210-5p in situ hybridization probe and immunofluorescence. The expression of miR-210-5p in CD31-labeled endothelial cells was significantly higher in the exosome-treated group than that in the Pbs group (Fig. 5e). Similar in vitro experiments were conducted to assess the expression of miR-210-5p in endothelial cells after OGD. The results indicated a non-significant increase in miR-210-5p expression, which is consistent with our previous qRT-PCR findings. However, miRNA expression significantly increased after the addition of pericyte-derived exosomes (Fig. 5f, g). These findings indicated a significant increase in miR-210-5p expression in pericytes under ischemia-hypoxic conditions. Moreover, miR-210-5p is transmitted to endothelial cells via exosomes and exerts distinct biological functions.

### Pericyte-derived exosomes improve Bend3 cell barrier function via miR-210-5p

**Fig. 6:**
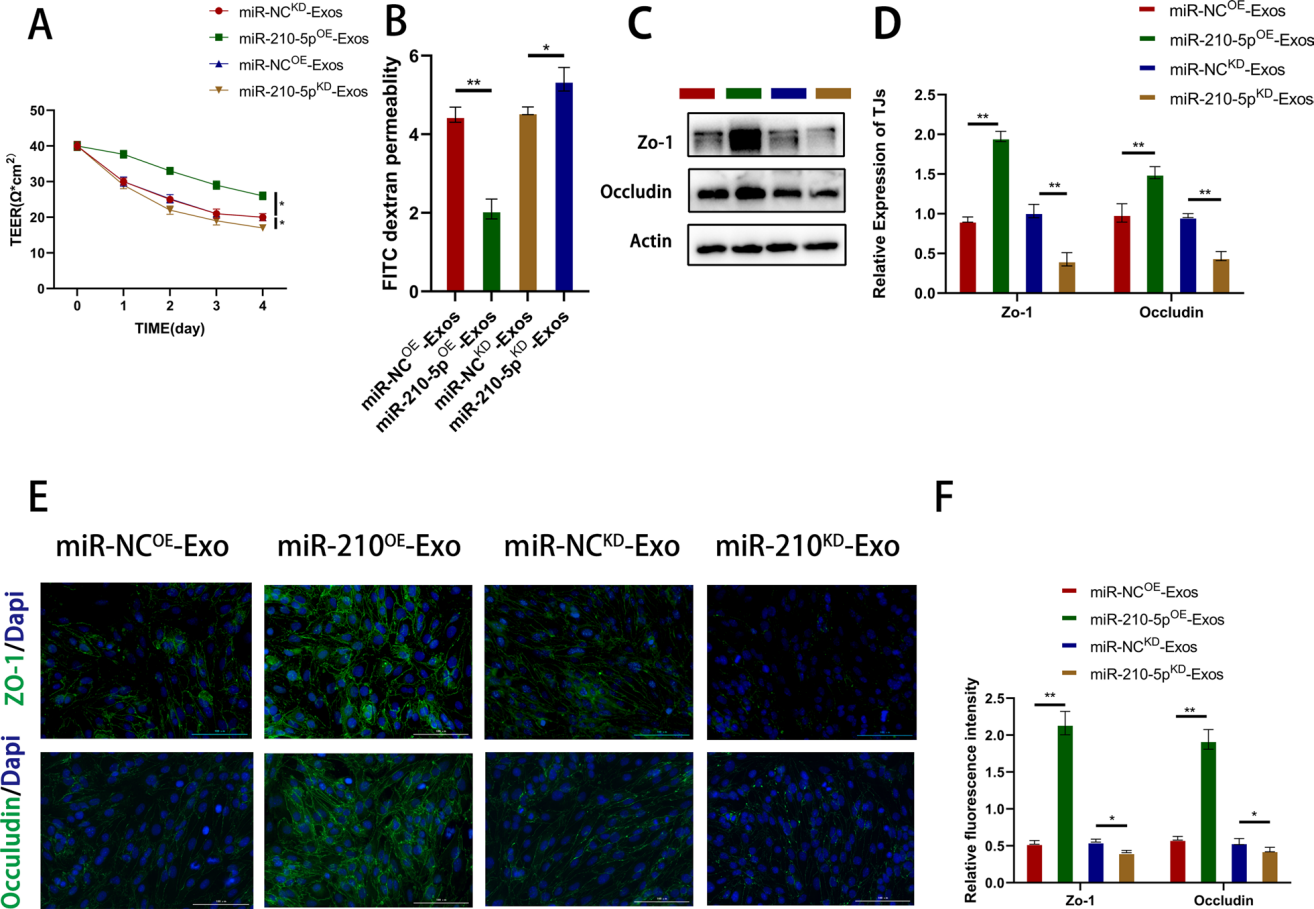
Pericyte-derived exosomes improve Bend3 cell barrier function via miR-210-5p. **A**, **B** The effect of exosomal miR-210-5p on cell barrier function was assessed by Teer value and FITC-dextran permeation assay. **C**, **D** Detection and quantification of TJs protein expression by immunoblotting. **E**, **F** Typical immunofluorescence staining images of ZO-1 and Occludin and quantification

Pericytes were modified using lentivirus-mediated overexpression (miR^OE^) or knockdown (miR^KD^) of miR-210-5p and were compared to their respective negative controls (miR-NC^OE^, miR-NC^KD^) to investigate the effect of exosomal miR-210-5p on BSCB following SCI. Transfection efficiency was assessed using qRT-PCR, followed by exosome isolation for subsequent investigations. Compared to miR-NC^OE^ exosomes, miR-210-5p^OE^ exosomes significantly upregulated miR-210-5p expression. Similarly, miR-210-5p^KD^ exosomes significantly upregulated miR-210-5p expression compared with miR-NC^KD^ exosomes. miR-210-5p expression in the target cells was consistent with the corresponding results. Subsequently, cell barrier function experiments were performed. After OGD of Bend3 cells, the Teer value of the miR-210-5p^OE^ group was significantly higher than that of the miR-NC^OE^ group. In contrast, the miR-210-5p^KD^ group showed contrasting results (Fig. 6a). Similar results were observed in FITC-dextran permeation experiments (Fig. 6b). For immunofluorescence staining, Bend3 cells were pre-exposed to OGD. We observed a significant increase in the expression of ZO-1 and Occludin in the miR-210-5p^OE^ group, whereas the fluorescence intensity in the miR-210-5p^KD^ group was significantly decreased (Fig. 6e, f). Immunoblotting analysis of TJ protein expression yielded similar results (Fig. 6c, d). These findings indicated that miR-210-5p expression contributes to the restoration of the Bend3 cell barrier function in vitro.

### Exosomes promote the amelioration of BSCB by delivering miR-210-5p

**Fig. 7:**
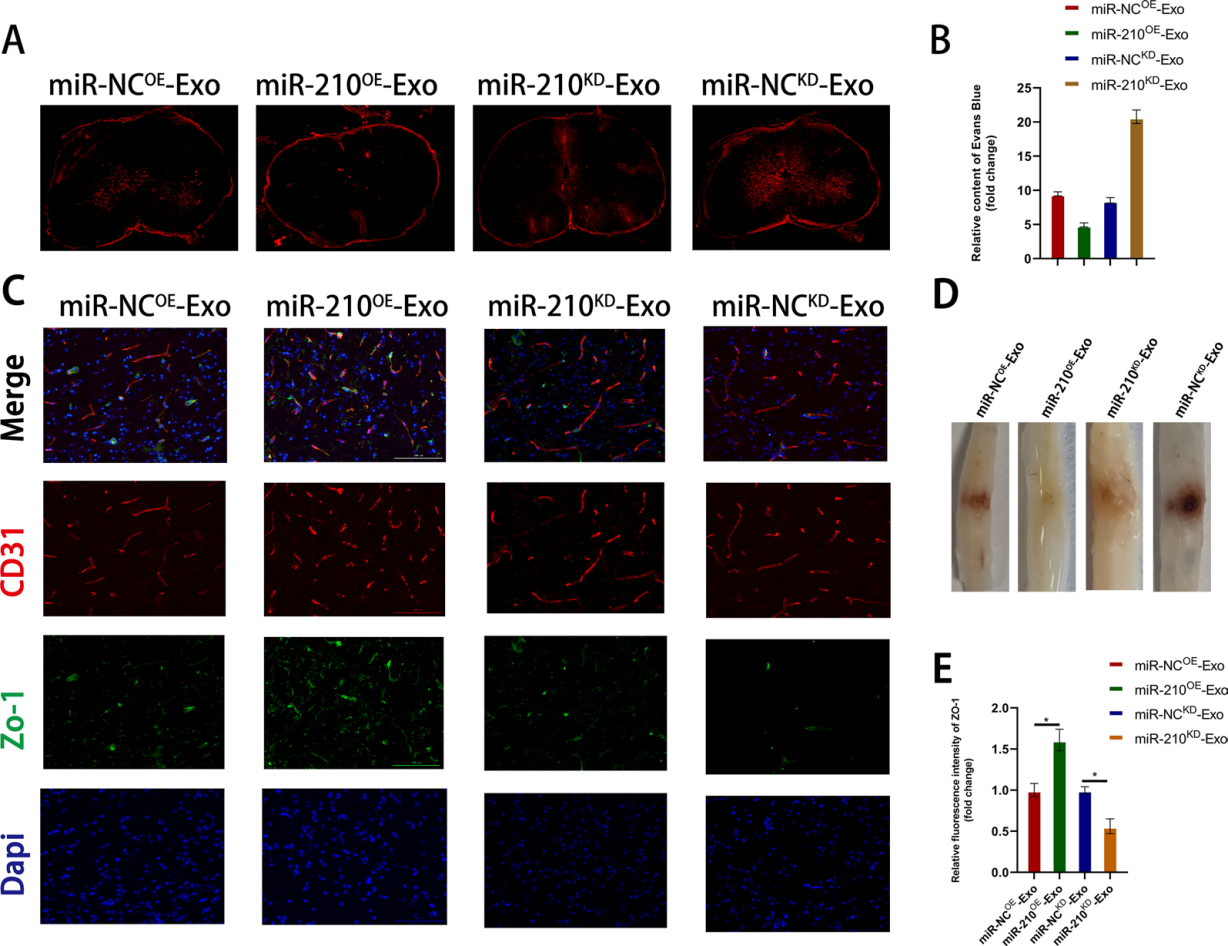
Exosomes promote amelioration of BSCB by delivering miR-210-5p. **A**, **B** Representative fluorescence images and quantification of spinal cord cross-sections from the injured area after EB injection 7 days after spinal cord injury. **C**, **E** Representative immunostaining images and quantification of Z0-1/CD31 in the spinal cord injury area. **D** Representative appearance of the spinal cord 7 days after spinal cord injury

We conducted several in vivo experiments to investigate the role of miR-210-5p in exosome-mediated functional recovery following SCI. First, fluorescence images were obtained of cross-sections of the injured area of the spinal cord after injecting EB 7 days post-injury. The miR-210^OE^ exosome group exhibited a significant decrease in EB infiltration compared to the miR-NC^OE^ group, whereas the miR-210-5p^KD^ group showed the opposite result (Fig. 7a, b). In addition, immunofluorescence staining revealed a significant increase in the expression of ZO-1 in the miR-210^OE^ treatment group compared to that in the miR-NC^OE^ group. Moreover, a substantial increase in colocalization with CD31 was observed, indicating an improvement in blood-spinal cord barrier function. However, the results of miR-210^KD^ exosome treatment were reversed (Fig. 7c, e). Spinal cord images showed similar results (Fig. 7d). Therefore, we speculated that miR-210-5p improves early barrier function via exosomes, reduces subsequent secondary cascades, and promotes the restoration of functional behavior following SCI.

### Exosomal miR-210-5p activates JAK1 by directly targeting the 3′-UTR

**Fig. 8:**
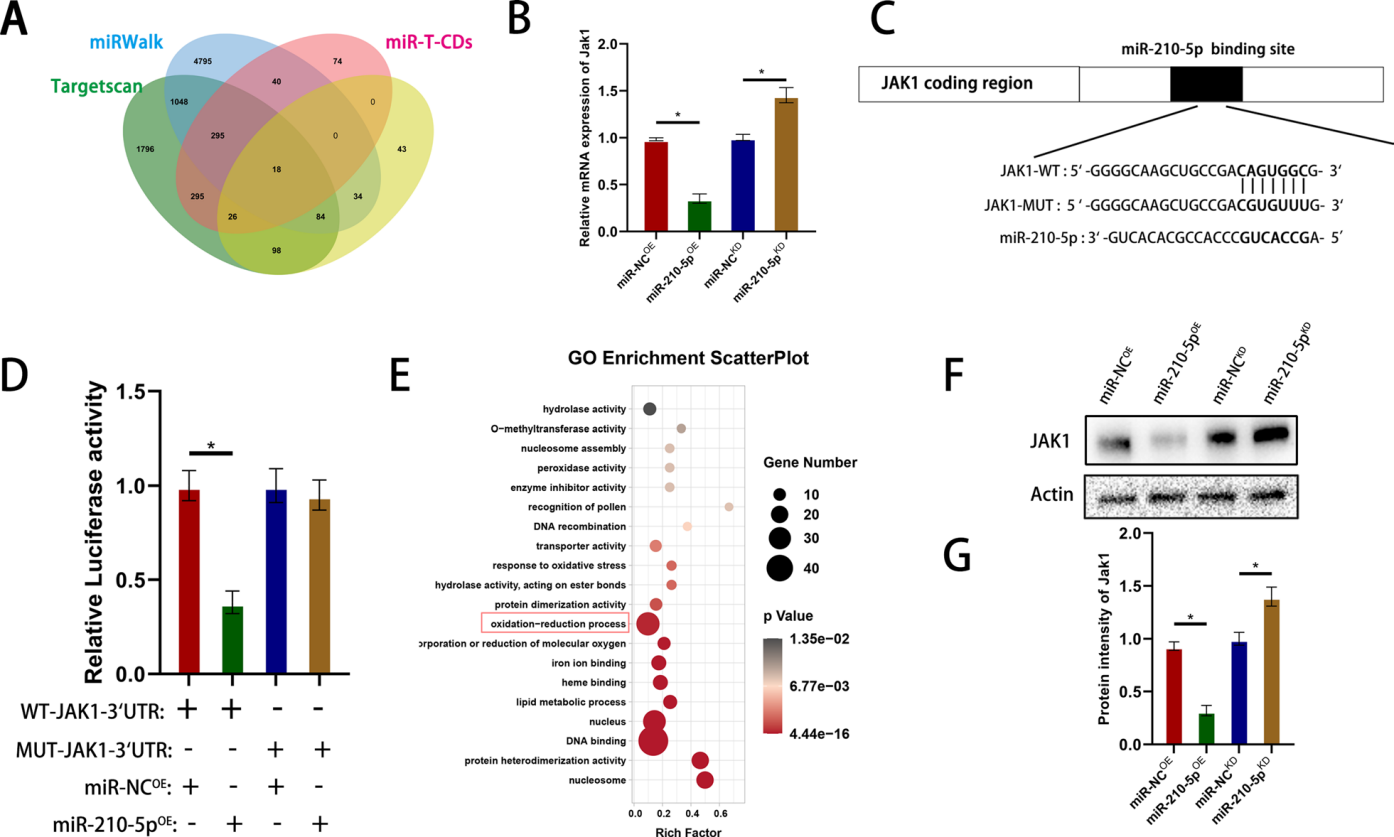
Exosomal miR-210-5p conditions JAK1 by directly targeting the 3′-UTR. **A** Predicted target genes of miR-210-5p. **B** qRT-PCR to verify the expression of target genes. **C** miR-210-5p regulates JAK1 by directly targeting the 3′UTR region. **D** Luciferase reporter assay confirmed that JAK1 is a target gene of miR-210-5p. **E** GO enrichment analysis of miR-210-5p target genes. **F**, **G** Western blot analysis of downstream target gene expression after miRNA overexpression and knockdown

We investigated the downstream genes of miR-210-5p to better understand the possible mechanism of exosomal miR-210-5p in repairing BSCB barrier function. We predicted 18 possible target genes using an online database of miRNA targets (Fig. 8a). qRT-PCR analysis revealed that miR-210 overexpression decreased JAK1 expression, whereas miR-210 knockdown upregulated JAK1 mRNA levels (Fig. 8b). Mutant and wild-type JAK1 3′-UTR sequences were constructed based on the predicted binding sites for miR-210-5p to validate its status as a direct target. Luciferase activity in Bend3 cells was significantly reduced when the wild-type 3′-UTR region of JAK1 was co-transfected with miR-210-5p compared to the control group. However, co-transfection with miR-210-5p and the MUT 3′-UTR region of JAK1 did not significantly inhibit luciferase activity (Fig. 8c, d). Western blotting further confirmed that miR-210 overexpression downregulated JAK1 expression, whereas miR-210 knockdown upregulated JAK1 protein levels (Fig. 8f, g). GO enrichment analysis of the predicted target genes revealed the potential involvement of mi-210-5p in hypoxia and oxidative stress responses (Fig. 8e). This is consistent with our previous hypothesis that miR-210-5p may be involved in the response to hypoxia and the hypoxic environment following injury.

### Exosomal miR-210-5p regulates target cell barrier function via the JAK1/STAT3 pathway

**Fig. 9:**
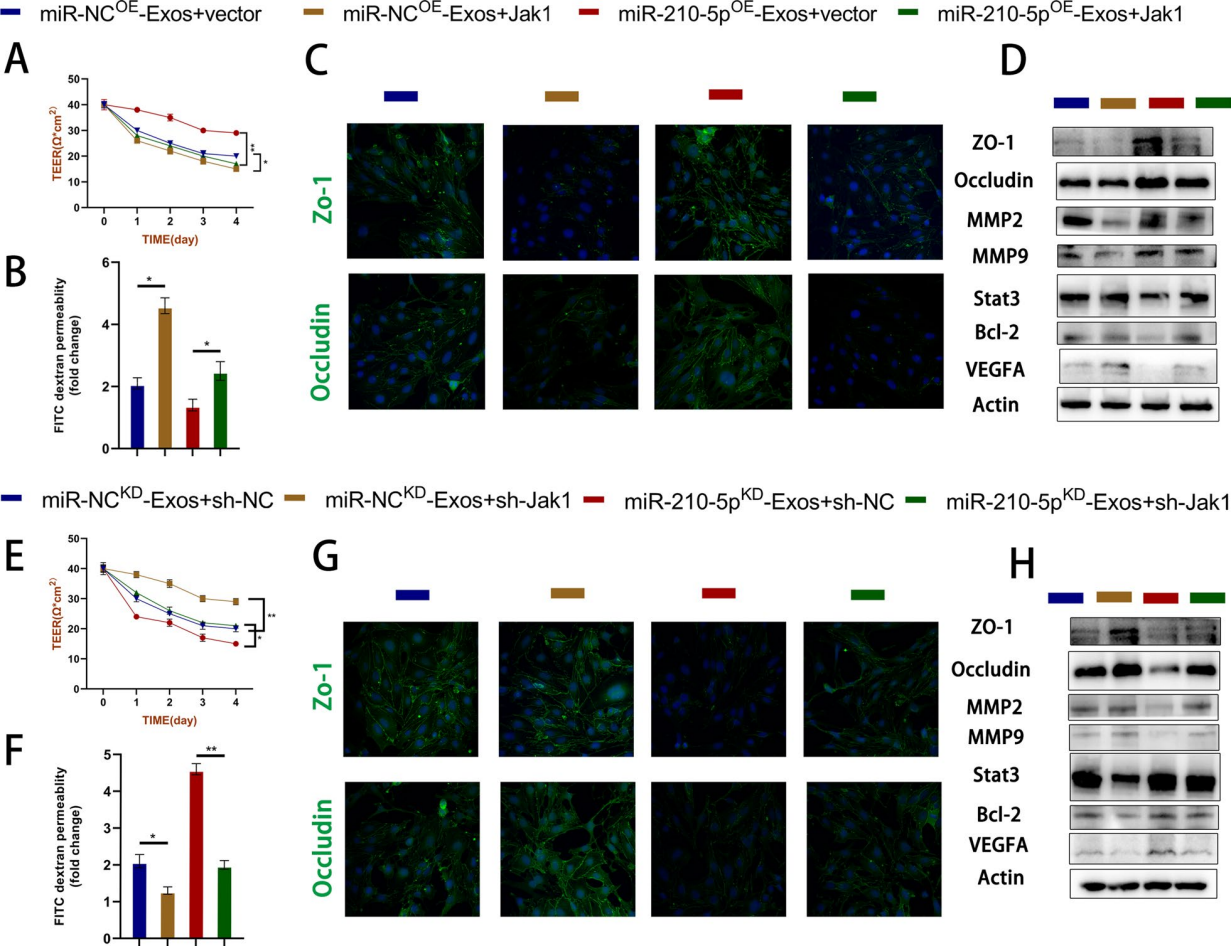
Exosomal miR-210-5p regulates target cell barrier function via JAK1/STAT3 axis. **A**–**H** A series of gain-of-function and loss-of-function experiments, including Teer value, FITC-dextran permeation assay, immunoblotting, and immunofluorescence, were used to verify the functional impact of the miR-210/JAK1/STAT3 axis on target cells. The expression level of TJs protein was detected by western blotting and immunofluorescence

We conducted gain-of-function and loss-of-function experiments to confirm the involvement of JAK1/STAT3 in the target cell barrier function. First, we introduced miR-210^OE^ and miR-NC^OE^ exosomes into JAK1-overexpressing Bend3 cells, and miR-210^KD^ and miR-NC^KD^ exosomes into JAK1-silenced Bend3 cells. The findings indicated that JAK1 overexpression increased FITC-dextran permeability and decreased the Teer value (Fig. 9a, b). Immunofluorescence experiments demonstrated that JAK1 overexpression reversed the protective effects of miR-210^OE^ exosomes on TJs, resulting in a reduced fluorescence intensity of ZO-1 and Occludin (Fig. 9c). Similar findings were also observed in the Western blot analysis (Fig. 9d). Furthermore, the adverse effects of miR-210^KD^ exosome administration were mitigated by silencing JAK1 (Fig. 9e–h). The JAK/STAT pathway is a crucial signaling pathway involved in cellular development and homeostasis. Recent studies have suggested that STAT3 may be involved in multiple mitochondrial functions, including electron transport chain regulation and ROS generation. Therefore, we simultaneously detected the transcription factor STAT3 downstream of JAK1 and measured its expression level using immunoblotting (Fig. 9d, h).

### Exosomes regulate lipid peroxidation in target cells via the miR-210/JAK1/STAT3 axis and improve mitochondrial function

**Fig. 10:**
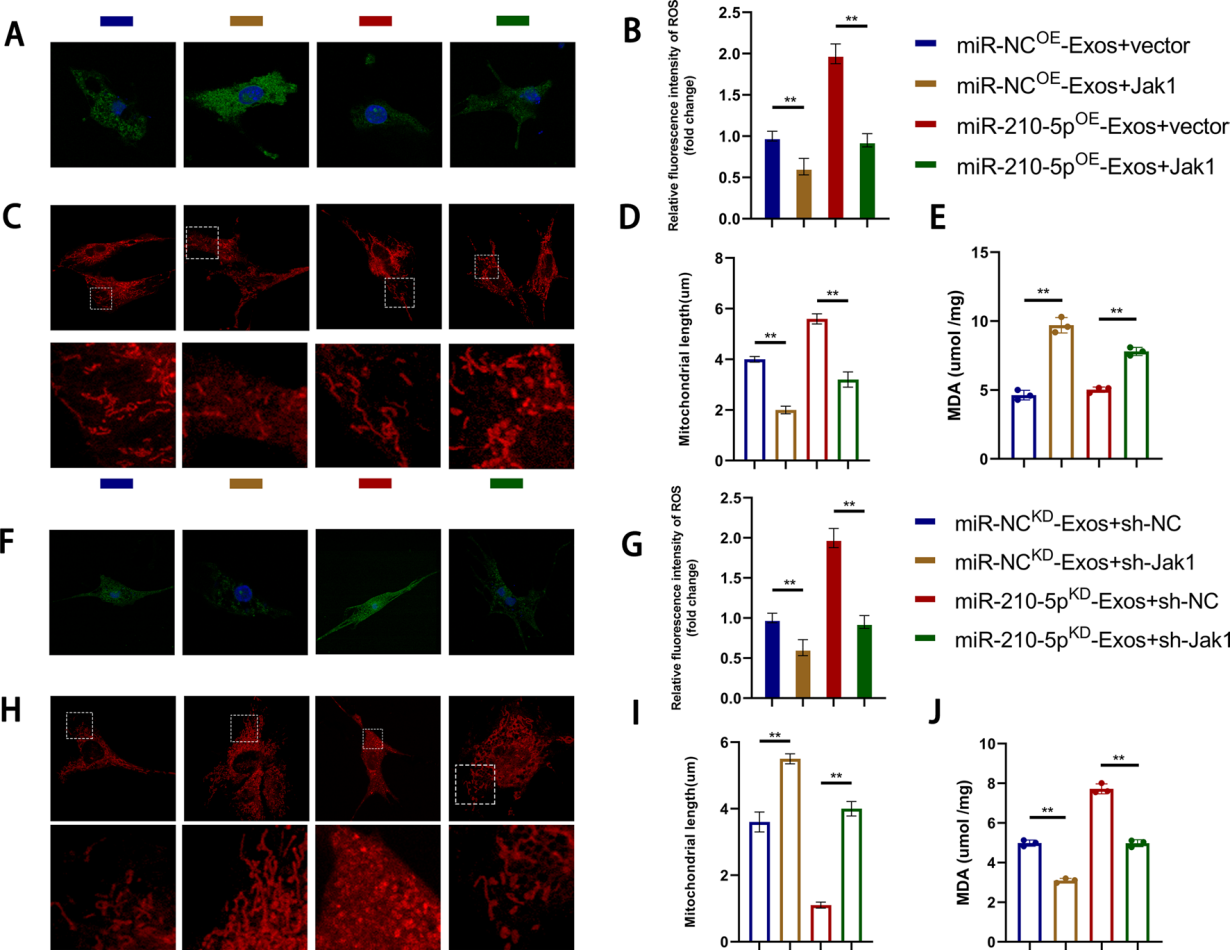
Exosomes regulate lipid peroxidation levels in target cells through the miR-210/JAK1/STAT3 axis and improve mitochondrial function. **A**–**J** The effects of the miR-210/JAK1/STAT3 axis on lipid peroxidation and mitochondrial function were validated in vitro through a series of gain-of-function and loss-of-function experiments. Immunofluorescence detection and quantification of lipid peroxidation levels in Bend3 cells. Quantitative detection of MDA (Malondialdehyde, MDA) content in Bend3 cells. Representative images of mitochondrial morphology under confocal microscopy and quantification of mitochondrial length

A series of in vitro experiments involving gain-of-function and loss-of-function approaches were conducted to investigate the regulation of mitochondrial function and the promotion of lipid peroxidation by miR-210/JAK1/STAT3. JAK1 overexpression reversed the effects of miR-210^OE^ exosomes, resulting in increased lipid peroxidation, reduced mitochondrial length, and increased fragmentation (Fig. 10a–d). We quantified the level of MDA in the target cells and presented the results (Fig. 10e). Furthermore, the adverse effects of miR-210^KD^ exosomes were reversed by silencing JAK1, leading to contrasting outcomes (Fig. 10f–j).

## Discussion

SCI is a severe traumatic condition that causes permanent neurological damage and is characterized by primary injury followed by a secondary cascade. The lack of effective treatments for SCI can be attributed to the inherent characteristics of the disease and its technical limitations. The use of glucocorticosteroids in clinical practice has been associated with numerous side effects [41, 42]. Exosomes possess significant potential as natural drug carriers in the central nervous system because of their ability to traverse the BBB [43]. MSCs have received significant research attention because of their differentiation potential and ability to release neuroprotective factors [44–46].

Pericytes play a crucial role in the development and maintenance of the BBB by coordinating the functions of endothelial cells, astrocytes, and oligodendrocyte progenitor cells [47]. Pericytes are also involved in the pathophysiology of neurodegenerative and cerebrovascular diseases [48–50]. Numerous studies have shown that endothelial cells and pericytes promote angiogenesis via direct physical interactions and release of paracrine molecules [51]. Impaired crosstalk between endothelial cells and pericytes can lead to dysfunction of BBB after SCI [25, 26]. However, whether SCI pericytes exert their effects through exosomes remains unclear. Previous research has indicated that pericyte-derived extracellular vesicles contain diverse cellular components, such as growth factors, miRNAs, and cyclic RNAs, among others [52]. In the present study, we found that pericyte-derived exosomes promoted restoration of BBB functionality.

Multiple studies have demonstrated that the disruption of the microvascular structure and BSCB plays a crucial role in secondary injury [9, 53, 54]. Following SCI, the spinal cord experiences a severe ischemia-hypoxic environment caused by microvascular destruction, spinal cord hemorrhage, and edema [55, 56]. An ischemia-hypoxic environment can stimulate angiogenesis, resulting in neovascularization in damaged areas during the early stages. However, these newly formed blood vessels lack tight junction proteins, rendering them highly permeable and unable to resist the influx of inflammatory factors caused by secondary damage. Previous research has demonstrated that vascular regeneration occurs within 2 weeks after SCI. This process is regulated by several chemicals and transcription factors, including the HIF-1α, SOX17, and VEGF pathways [57–60]. HIF-1α expression in the spinal cord tissue was also validated post-injury, reflecting the early hypoxic environment inside the spinal cord tissue. Therefore, repairing the BSCB is of great significance crucial for alleviating secondary injuries and promoting functional recovery following SCI.

Blood vessels are damaged within 30 min of SCI [61]. Severe ischemia and hypoxia are primary causes of early endothelial cell death. ROS-induced lipid peroxidation is a critical mechanism that leads to subsequent endothelial cell death [62]. Several researchers have developed specific neuroprotective drugs that target the lipid peroxidation process [39, 63]. Therefore, our study on microvascular and endothelial cells primarily focused on oxidative stress, including lipid peroxidation, mitochondrial status, and malondialdehyde content. Immunofluorescence staining and MDA quantitative analysis revealed that miR-210-5p overexpression effectively inhibited lipid peroxide production and protected the mitochondrial function in target cells. Dysregulation of ROS production can cause microvascular damage and endothelial cell dysfunction. Many researchers have aimed to develop antioxidant therapeutic strategies targeting endothelial cells to improve mitochondrial and vascular functions [64–67]. Researchers have also investigated antioxidant therapeutic strategies targeting the barrier function in other degenerative diseases of the central nervous system, including Alzheimer's and Parkinson's diseases [68–70].

Based on our findings, we investigated the potential mechanisms underlying the protective effects of pericyte-derived exosomes in SCI. Exosomes facilitate the targeted delivery of specific miRNAs to mediate specific biological functions in target cells. Their composition and quantity are altered in response to hypoxia [71–73]. miRNA sequencing revealed significant upregulation of miR-210-5p in ischemic and hypoxic pericytes. Furthermore, we found that this miRNA could be transferred to endothelial cells via exosomes. Previous studies have primarily focused on the role of miR-210-5p in cancer, diabetes, Parkinson's disease, and other conditions, but its involvement in SCI remains unexplored [74–76]. MiR-210-5p was upregulated under hypoxic conditions in a study of hepatocellular carcinoma, which complied with our sequencing results and in situ hybridization probes [77]. Pericytes respond to hypoxia by undergoing changes in morphology and transcriptome [78, 79]. Therefore, pericytes and miR-210-5p may cooperate during angiogenesis in an early hypoxic environment following SCI. A series of gain-of-function and loss-of-function experiments identified JAK1/STAT3 as the downstream target of miRNAs. STAT3, a conventional signal transduction molecule, regulates oxidative stress and actively participates in the preservation of mitochondrial function [80, 81]. Our study reveals a mechanism of endothelial-pericyte interaction that contributes to the maintenance of BBB/BSCB integrity. These findings could potentially establish novel therapeutic strategies for SCI.

## Conclusion

Spinal cord injury remains a challenging condition because of the lack of effective treatment options. Exosomes have gained attention as potential therapeutic agents in recent years because of their distinctive attributes. Pericytes are crucial for the development and maintenance of the blood–brain barrier. Numerous studies have demonstrated that endothelial cells and pericytes promote angiogenesis through physical interactions and release of paracrine molecules. We hypothesized that pericyte-derived exosomes may promote the restoration of the BBB in SCI. This study showed that pericyte-derived exosomal miR-210-5p can regulate endothelial barrier function by inhibiting JAK1/STAT3. This regulatory mechanism involves modulation of lipid peroxidation and improvement of mitochondrial function. Our study revealed a part of the mechanism underlying endothelial-pericyte interaction that contributes to the maintenance of BBB/BSCB integrity. This finding could potentially lead to the development of novel therapeutic strategies for SCI.

## Materials and methods

### Primary cell culture and cell lines

Mouse brain microvasculature-derived pericytes were obtained from newborn mice using established protocols [82]. Following dissection and dissociation of the cerebral cortex, it was cut into 1 mm^3^ cubes using cold DMEM (C11965500BT, Gibco, USA). The cubes were digested by shaking at 37 °C for 1.5 h in DMEM supplemented with collagenase type 2 (1 mg/ml, BioFroxx) and DNase (15 μg/ml; Roche). Cells were obtained via centrifugation in 20% BSA (bovine serum albumin)/DMEM solution at 1000 × g for 20 min. The resulting cell pellet was digested in DMEM containing collagenase-dispase (1 mg/ml; Roche) at 37 °C for 1 h. Microvessel fragments were plated on a 33% continuous Percoll gradient (GE Healthcare, USA) after low-speed centrifugation. Following an additional centrifugation step, the upper layer, which was enriched in microvessels, was collected. Microvascular fragments were cultured in pericyte medium (ScienCell, USA) for 2 weeks, resulting in the isolation of purified pericytes. Cell purity was determined using immunofluorescence staining for α-SMA (Cell Signaling Technology Cat# 19245, RRID:AB_2734735) and PDGFRβ (Cell Signaling Technology Cat# 45176, RRID:AB_2924258).

The Bend3 (brain-derived Endothelial cells. 3) endothelial cell line was obtained from the Shanghai Cell Bank of the Chinese Academy of Sciences. Cells were cultured in DMEM (high sugar) supplemented with 10% fetal bovine serum (Gibco, USA) and 1% penicillin/streptomycin.

### Construction of in vitro BBB model

The BBB model was established by culturing the cells in Transwell inserts (Cat# 3450, Corning, USA) [83]. Bend3 endothelial cells were plated at a density of 1.5 × 10^5^ cells/cm^2^ on the upper side of the insert and in the wells of 12-well culture plates, with and without pericytes. Once the cells adhered tightly in vitro, the establishment of the BBB model was confirmed and designated as day 0.

### Oxygen glucose deprivation (OGD)

Primary pericytes were cultured under hypoxic conditions with 1% O_2_ for 24 h in glucose-free DMEM before subsequent experimentation. The constructed BBB model was subjected to OGD for 3 h before conducting additional experiments such as Teer value assays.

### Exosome isolation and identification

Exosomes were isolated from supernatants of normal cultured and OGD-exposed pericytes [84]. When pericytes reached 80% confluence, the medium was replaced with exosome-free medium (Cat# 41210ES76, Yeasen, China). After 24-h period, the medium was collected and centrifuged at 300 × g for 10 min, followed by an additional centrifugation step at 2000 × g for 10 min. Cell debris were eliminated using a 0.22 μm filter (Cat# SLGV033R, Millipore, USA) after centrifugation. The supernatant was transferred to ultra-clear tubes (Cat# 12706558, Beckman, USA) and ultracentrifuged at 110,000 × g (Beckman, XPN-100) for 90 min at 4 °C. The pellet was resuspended in PBS, and ultracentrifugation steps were repeated. The purified exosomes were resuspended in PBS and either used immediately for the next experiment or stored at − 80 °C for future use. Exosome surface-specific markers, including CD9 (BD Biosciences Cat# 551808, RRID:AB_394262), CD63 (Santa Cruz Biotechnology Cat# sc-15363, RRID:AB_648179) and CD81 (RayBiotech Cat# DS-MB-00892, RRID:AB_852749), were detected using Western blotting. Transmission electron microscopy (USA, Tecnai G2) was used to observe the morphology of the obtained exosomes. Nanoparticle tracking assays (NTA) were used to determine the concentration and size range of exosomes.

### miRNA microarray assay

MicroRNA arrays were performed by the BGI Company (Wuhan, China) to analyze pericytes induced by OGD and the control groups. Each group contained three samples. Microarray analysis was conducted using the Affymetrix miRNA 4.0 platform (Santa Clara, CA, USA). Following normalization, log2 transformation, and probe annotation, we identified differentially expressed genes (DEGs) based on the criteria of |log2 fold change|> 1.5 and adjusted *P* value < 0.05. Hub DEGs were selected for further investigation.

### Exosomes uptake by Bend3 cells

Exosomes were incubated with fluorescent probes (Cat# C1991S, Beyotime, China) for cell membrane labelling. Following incubation, excess dye was removed by ultracentrifugation and the exosomes were washed three times. After incubating the fluorescently labeled exosomes with Bend3 cells for 24 h, cells were washed with PBS and fixed with 4% paraformaldehyde. Subsequently, exosome uptake by Bend3 cells was observed using fluorescence microscopy.

### Vector construction and cell transfection

The in vitro compensation assays were validated using lentiviral vectors [85]. We used LV2-MMU-miR-210-5p-Simic, LV2-MMU-miR-210-5p-Inhibitor, shRNA-targeted vectors, and a negative control vector constructed with an empty vector. Cells at 50% confluence were infected with lentiviral vectors at an appropriate multiplicity of infection (MOI), and the corresponding exosomes were subsequently extracted for further experiments.

### RNA extraction and qRT-PCR

An RNA-Quick Purification Kit (Esunbio, China) was used to extract total RNA from the cells and exosomes. A reverse transcription system was used to synthesize cDNA for miRNAs and mRNA. qRT-PCR assays were subsequently performed on an ABI Steponeplus™ Real-time PCR system using the SYBR Green PCR master mix kit. miRNA and mRNA expression levels were evaluated using U6 and GAPDH as internal controls, respectively. The relative expression was determined using the 2^–△△^CT method.

### Western blot assay

Cell and tissue proteins were extracted using commercially available kits (Cat# KGP2100, KeyGene, China). The protein concentration was determined using the BCA method (Cat# 23225, Thermo Fisher Scientific, USA). Proteins were separated using SurePAGE™ gels (Genescript, China) and transferred onto polyvinylidene fluoride (PVDF) membranes. The membranes were blocked with 5% BSA blocking solution for 1 h at room temperature, followed by overnight incubation with primary antibodies (1:1000) at 4 °C. The antibodies used included MMP-2 (Santa Cruz Biotechnology Cat# sc-58386, RRID:AB_784596), MMP-9 (Santa Cruz Biotechnology Cat# sc-13520, RRID:AB_627961), STAT3 (Santa Cruz Biotechnology Cat# sc-8019, RRID:AB_628293), Bcl-2 (Santa Cruz Biotechnology Cat# sc-16323-R, RRID:AB_653044), VEGFA (Abcam Cat# ab267566, RRID:AB_2927434), Actin (Santa Cruz Biotechnology Cat# sc-81178, RRID:AB_2223230). The following day, the samples were washed with TBST and incubated with the appropriate secondary antibodies (1:10,000; Cat#A0216, Cat# A0208, Beyotime) for 2 h. The resulting bands were visualized using a gel imager (ChemiDoc XRS, USA) and an ECL reagent (Thermo Fisher Scientific).

### Immunofluorescence staining

Frozen tissue sections or cells were fixed with 4% paraformaldehyde, permeabilized with 0.3% triton solution, blocked with 5% BSA solution, and incubated overnight at 4 °C with primary antibodies (1:200), including ZO1 (Thermo Fisher Scientific Cat# 33-9111, RRID: AB_2533148), CD31 (BD Biosciences Cat# 553369, RRID:AB_394815), α-SMA, PDGFRβ, Occludin (Proteintech Cat# 27260-1-AP, RRID:AB_2880820). After 1 h of incubation with appropriate secondary antibodies, DAPI (1:1000) staining was performed the following day. The fluorescence images were captured using a fluorescence microscope. The fluorescence intensity was quantified using ImageJ software.

### Luciferase reporter gene assay

Sequences corresponding to the 3′-UTR of JAK1 mRNA were synthesized by Ribio (Guangzhou, China). These sequences were cloned into the pGL3 luciferase reporter vector downstream of the coding region to construct the JAK1 3′-UTR reporter gene vectors (pGL3WT-JAK1 and pGL3-mut-JAK1). Bend3 endothelial cells were cultured in 96-well plates and co-transfected 24 h later with a reporter vector (100 ng/ml) and miR-210-5p^OE^ or NC mimics [5 nmol] using lipo2000. Firefly and Renilla luciferase signals were quantified after 72 h using a dual-luciferase assay kit (Promega).

### Morphological analysis of the mitochondria

Bend3 live cells were incubated with Mito-Tracker Red CMXRos (Cat# C1049B, Beyotime) working solution at 37 °C for 30 min, following the manufacturer’s instructions. The solution was subsequently removed, and pre-warmed cell culture medium was added. Images were acquired using confocal microscopy, and mitochondrial morphology was quantified with image J software.

### Determination of lipid peroxidation

Cellular lipid peroxidation levels were evaluated using the Click-iT Lipid Peroxidation Imaging Kit (C10446, Thermo Fisher Scientific), following the manufacturer's instructions. A Lipid Peroxidation MDA Assay Kit (Cat# S0131S, Beyotime, China) was used for the quantitative measurement of cellular lipid peroxidation levels by detecting lipid oxide (MDA) levels.

### Evaluation of BSCB permeability

#### Detection of barrier integrity using FITC-dextran

Cells were seeded onto inserts and measured upon reaching a confluent monolayer. FITC-dextran medium (100 μl, 1 mg/ml) was added to the upper compartment of the insert and 500 μl of the medium was added to the lower compartment. After incubation for 1 h, the fluorescence intensity of the underlying solution was measured using a spectrofluorometer.

#### Transepithelial electrical resistance (TEER) measurements

Bend3 cells were seeded at a density of 1.5 × 10^5^ cells/cm^2^ on transwell inserts with a pore size of 0.4 μm and cultured to a confluent monolayer for measurement. Before each measurement, the cells were pre-equilibrated with Hank’s Balanced Salt Solution (HBSS) for 30 min, and resistance was measured using a Millicell^®^ ERS instrument (Millipore, USA).

TEER (Ω cm [2]) = [TEER measurement − TEER control] × membrane area. The TEER control value referred to the TEER measurement obtained when the insert was cell-free.

### Preparation of the contusive SCI mouse model and experimental groups

Animal experiments and operations were approved by the Ethics Committee of Animal Experiments at the First Affiliated Hospital of Nanjing Medical University. SCI models used in our experiments were prepared as previously described [86]. Mice were anesthetized using isoflurane inhalation. The lamina was excised at T8 vertebral level to expose the spinal cord. A rod (weighing 5 g) was then dropped onto the spinal cord from a height of 6.5 cm using a spinal cord impactor (68097; RWD, USA). Bladder function was initially impaired following SCI, necessitating assistance with urination twice daily until the bladder function was restored. Immediately after the completion of SCI in mice, PBS (200 μl) or exosomes (200 μg total exosomal protein in 200 μl PBS) were injected into the tail vein according to the experimental requirements.

### Design of the animal experiments

The sample size of four mice was determined based on the success rate of the animal model. Each group, whether experimental or control, consisted of at least four mice. Three mice died from post-surgical hemorrhage during the experiment. Our final assessment of motor function was as of 28 days after SCI, as previous studies have indicated that motor function in mice stabilizes 28 days following SCI. Following SCI in mice, the BBB was immediately disrupted and stabilized after 14 days. Therefore, we assessed the permeability of the BBB by Evans blue 7 days after SCI.

### Functional test evaluation

#### Basso mouse scale (BMS) analysis

The restoration of lower-limb motor function in mice was assessed using the BMS method. The scores were evaluated based on various criteria, including lower limb joint mobility, coordination, paw posture, trunk stability, and tail posture. The scores were measured preoperatively and at various time points (1, 3, 7, 14, 21, and 28 days) after SCI.

#### Swimming test

Motor function recovery following SCI in mice was evaluated using the swimming test. Mice were placed in a tank and trained to swim from side to side. The researchers then assessed the mice's forelimb dependence, hindlimb movement and alternation, body angle, and trunk stability using the Louisville Swimming Scale. Each mouse was tested twice and the average score was used as the final score.

#### Footprint analysis

The gait and motor coordination of the mice 28 days after SCI were assessed using footprint analysis. Different dyes were used to stain the forelimbs (blue) and hindlimbs (red) of mice. When the mice walked in a straight line at constant speed, the resulting patterns were captured using a digital camera, and a subset of representative images was selected for analysis.

### Evaluation of the BSCB permeability

#### Nissl staining of spinal cord sections

Spinal cord tissue sections from different groups were subjected to Nissl staining on the 28th day following SCI in mice. Nissl's stain, characterized by a blue-purple color, is commonly used to visualize basic neural structures of the brain or spinal cord. Nissl bodies are sensitive indicators of the extent of nerve cell damage. Researchers blinded to the experiments were selected to observe the staining in the damaged area using an inverted microscope.

TEM observation of the ultrastructure of spinal cord microvessels.

On the 28th day post-SCI, mice were anesthetized and a section (2 mm × 2 mm) of the injured area of the spinal cord was selected. Spinal cord tissue was fixed in an electron microscope fixative solution for 2 h at room temperature, dehydrated using acetone, embedded in epoxy resin, and sliced into ultrathin sections. The morphology of the vascular tight junctions in the spinal cord was examined using TEM, and images were recorded.

#### Evans blue dye assays

On the 7th day following SCI, mice in each group were injected with 2% Evans Blue (EB) solution via the tail vein at a dose of 4 ml/kg. After 2 h, the anesthetized mice were perfused with normal saline. Next, the spinal cord from the injured area of the mouse was extracted, homogenized with 50% trichloroacetic acid solution, and centrifuged at 20,000 × g for 20 min. The supernatant was diluted 1:3 with ethanol, and the fluorescence intensity of EB was measured at an excitation wavelength of 620 nm and an emission wavelength of 680 nm.

### Statistical analysis

Experiments were performed with at least three independent biological replicates. Data are presented as the mean ± standard deviation. Data were analyzed using GraphPad software 8.0. Student's *t*-test was used for two-group comparisons, whereas one-way or two-way ANOVA was used for multivariate analysis. *P*-values < 0.05 were considered statistically significant.

## Data Availability

The datasets used and/or analyzed during the current study are available from the corresponding author on reasonable request.

## Abbreviations

BBB: Blood–brain barrier
Bend3: Brain-derived Endothelial cells. 3
BMS: Basso mouse scale
BSA: Bovine serum albumin
BSCB: Blood spinal cord barrier
EB: Evans blue
Mda: Malondialdehyde
NTA: Nanoparticle tracking assay
OGD: Oxygen glucose deprivation
SCI: Spinal cord injury
TEER: Transepithelial electrical resistance
